# An overview of the International Consensus Statement on achondroplasia

**DOI:** 10.1186/s13023-025-04189-y

**Published:** 2026-01-20

**Authors:** Inês Alves, Svein Otto Fredwall, Michael Hughes, Penelope J. Ireland, Morrys C. Kaisermann, Ravi Savarirayan

**Affiliations:** 1 ANDO Portugal, Évora, Portugal; 2 Comprehensive Health Research Centre (CHRC), Health and Sport Department, School of Health and Human Development, Évora University, Évora, Portugal; 3 National Centre for Rare Diseases, Sunnaas, Nesodden Norway; 4 Biotech Industry Liaison Committee Chair, Little People of America, Sonoma, CA USA; 5 School of Health and Rehabilitation Sciences, University of Queensland, Brisbane, Queensland Australia; 6 Queensland Children’s Hospital, Brisbane, Queensland Australia; 7 Growing Stronger, Saratoga, CA USA; 8 Fundación ALPE Acondroplasia, Gijón, Spain; 9 Murdoch Children’s Research Institute, Royal Children’s Hospital Melbourne, University of Melbourne, Parkville, Victoria Australia

**Keywords:** Achondroplasia, Consensus statement, Guidelines, Multidisciplinary care, Patient authors, Patient care team, Perspectives, Psychosocial support systems, Short stature

## Abstract

Achondroplasia is the most common form of short-limbed short stature of genetic origin. Most people with achondroplasia live fully independent, productive, and socially engaged lives. However, the condition is associated with several potential medical complications. Individuals with achondroplasia may experience medical, functional, and psychosocial challenges at different times in their life. The goal for lifelong care of individuals with achondroplasia is to optimize their physical and mental health through provision of individualized care and to promote participation and inclusion in society. In this article, as a tool for individuals with achondroplasia, their families, and their healthcare team, we provide a guided overview of the 2022 International Consensus Statement for management and care of individuals with achondroplasia. The International Consensus Statement recommendations are based on current, best available knowledge. We provide commentary on the recommendations from the perspective of both patients and physicians through addressing medical/developmental considerations, the healthcare system, and psychosocial considerations.

**Supplementary information:**

The online version contains supplementary material available at 10.1186/s13023-025-04189-y.

## Plain Language summary

Achondroplasia is the most common form of short stature with shortened arms and legs. Achondroplasia is rare and often requires lifelong care by multiple team members. The aim of medical care for people with achondroplasia is to optimize health and promote participation in society. The 2022 International Consensus Statement, written by expert doctors, therapists, and patient representatives, is a complex and detailed document developed for healthcare professionals. In this article we provide people with achondroplasia and their families with the most current and accurate information based on the International Consensus Statement. We also provide comments from our own viewpoints as people with achondroplasia, family members, doctors, therapists, and other people involved in health care. The article covers (1) medical and developmental considerations from infancy to adulthood, (2) mental health and social support, and (3) the healthcare system. The article also includes four illustrations that show the main recommendations for medical care of babies, children, teenagers, and adults with achondroplasia.

## Background

Achondroplasia, the most common form of short-limbed short stature [1–3], is a rare condition caused by a genetic mutation in the *FGFR3* gene, resulting in impaired overall bone growth [2]. About 80% of babies born with achondroplasia have parents of average height. Achondroplasia may be diagnosed before or after birth. The characteristic features of achondroplasia include an average-sized torso but disproportionately short arms and legs, enlarged head with a prominent forehead, and reduced nasal bridge [2], (Box 1). Potential complications associated with achondroplasia include foramen magnum compression (compression on the spinal cord at the base of the skull), sleep apnea, spinal stenosis (tightness in the spinal canal) and deformities, bowing of the legs, recurrent ear infections, and leg and back pain [1, 2] (Box 1).

**Box 1:** Characteristic features and complications of achondroplasia

**Clinical features** • Short stature • Disproportionately shortened limbs • Frontal bossing (prominent forehead), underdeveloped facial skeleton with depressed nasal bridge (sunken nose) • Relatively enlarged head (head circumference > 97th percentile) • Narrow chest • Trident-shaped hands with short fingers • Increased joint laxity (flexible/mobile joints) • In infants: thoracolumbar kyphosis (spinal curvature in middle and lower back) • In older children and adults: exaggerated lumbar lordosis (inward curvature of lower back), tibial bowing (bowed legs) **Common complications, with estimated prevalence if known** [2, 4–9] • Foramen magnum stenosis with cervicomedullary compression (20%) – see Box 3 • Sleep apnea (48–75%) • Recurrent middle ear infections (80–90%) • Symptomatic lumbar spinal stenosis (50–68%) • Tibial bowing (33–50%) • Dental malocclusion ( > 50%) • Conductive hearing loss (children: 17%; adults: 40%) • Enlarged adenoids/tonsils requiring adenotonsillectomy (25–40%) • Speech delay (20%) • Progressive thoracolumbar kyphosis (10–15%) • Symptomatic hydrocephalus requiring treatment (3–5%) See Fig. A for a visual guide to locations of common complications

**Fig. A:**
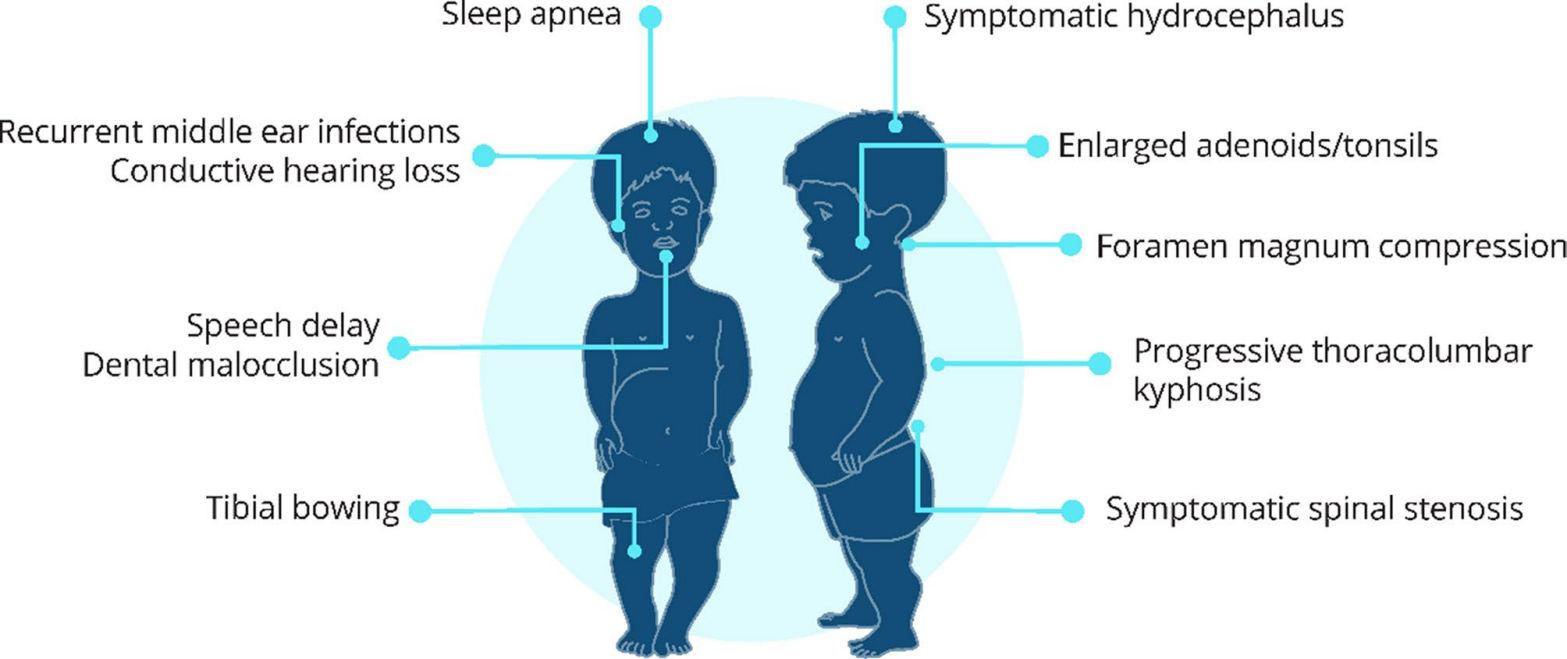
Schematic diagram of locations of common complications of achondroplasia

Most individuals with achondroplasia can be fully independent and have productive and socially engaged lives. However, individuals with achondroplasia may experience medical, functional, and psychosocial challenges, which can vary from very mild to very complex, at different points throughout their life. Environmental and societal constructions are significant factors contributing to day-to-day challenges faced by people with achondroplasia. Recommendations for management and care of individuals with achondroplasia were detailed in a 2022 International Consensus Statement, developed by 55 international experts from 16 countries and five continents [1]. The International Consensus Statement represents the first global effort to standardize care across the lifespan of people with achondroplasia. The recommendations of the International Consensus Statement address the key challenges and optimal management throughout life and across diverse medical specialty areas. The recommendations are based on current, best available knowledge and aim to support people with achondroplasia and their families to minimize medical complications and pain, and promote independence and participation.

**Fig. 1:**
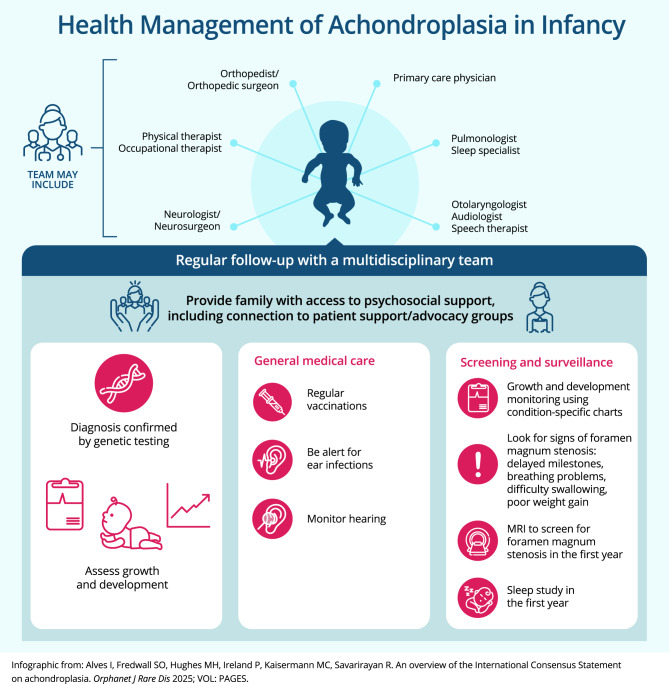
Key recommendations for care and management of achondroplasia in infants (infographic). MRI: magnetic resonance imaging

**Fig. 2:**
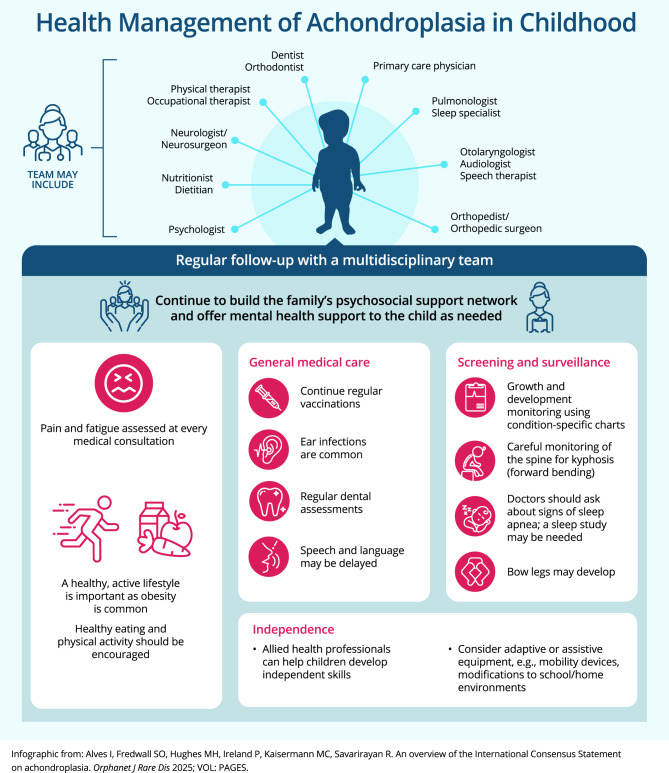
Key recommendations for care and management of achondroplasia in children (infographic)

**Fig. 3:**
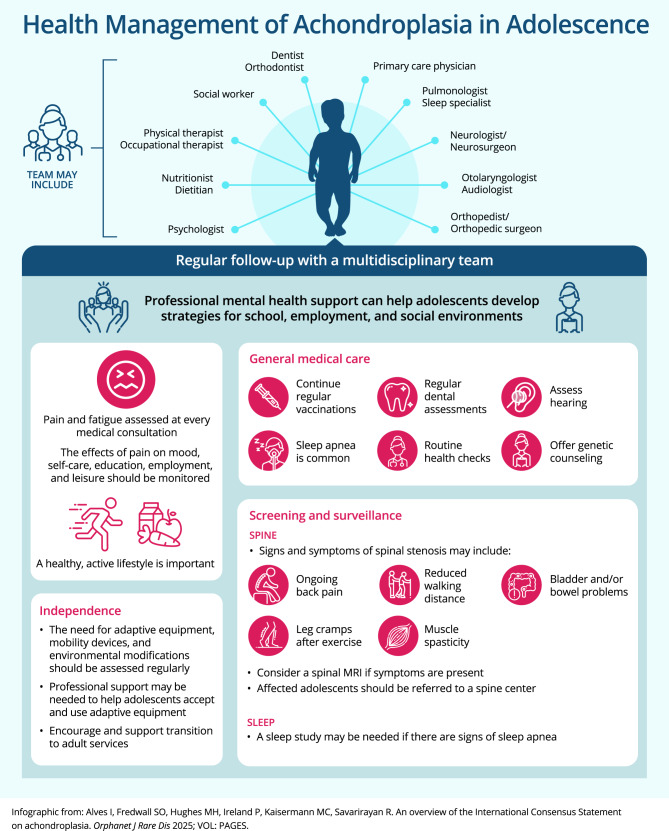
Key recommendations for care and management of achondroplasia in adolescents (infographic). MRI: magnetic resonance imaging

**Fig. 4:**
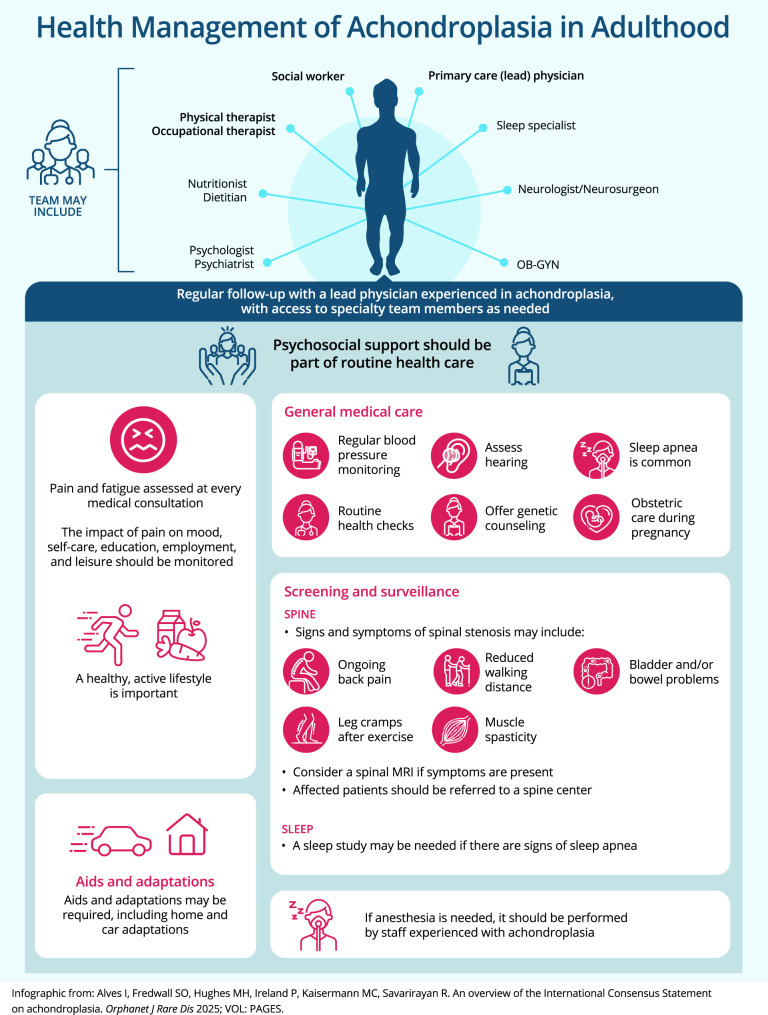
Key recommendations for care and management of achondroplasia in adults (infographic). MRI: magnetic resonance imaging; OB-GYN: obstetrician-gynecologist

In this article, we provide an overview of the International Consensus Statement. While the document is primarily intended for individuals with achondroplasia and their families, it also provides a useful starting resource for healthcare professionals (HCPs) without prior experience in achondroplasia. Three members of our author team provide their perspective as “patient authors”; in the context of authorship, we consider “patient” to be a broad term covering any person who has achondroplasia or has close life experience with achondroplasia, such as caregivers, family members, and members of patient advocacy groups [10]. In addition, we provide perspectives from three HCPs with expertise in clinical management of individuals with achondroplasia. We have addressed the recommendations of the International Consensus Statement within/across three broad categories: medical/developmental considerations, the healthcare system, and psychosocial considerations. Many topics span all categories, and the reader is encouraged to refer to all sections of the article. In the medical/developmental section, we address issues for the different life stages of infancy, childhood, adolescence, and adulthood, illustrated as infographics in Figs. 1, 2, 3, and 4, respectively. We have also included plain language callout boxes, including a glossary of terms (Box 2), intended for use by patients, caregivers, and non-expert HCPs. Additional guidance on specific topics (patient advocacy organizations, growth and development, positioning of infants/children, weight control) is available in Additional files 1–4. The relevant recommendation numbers from the International Consensus Statement are cited where appropriate.

**Box 2:** Glossary of terms

**Adenotonsillectomy:** surgical removal of adenoids and tonsils **Apnea:** temporary stoppage of breathing (often during sleep) **Clonus:** involuntary muscle spasms **Cervicomedullary compression:** compression of the spinal cord at the base of the skull **Foramen magnum:** the opening at the base of the skull housing the spinal cord **Genu varum:** bowed legs **Homozygous:** having two copies of a gene **Hydrocephalus:** an abnormal buildup of cerebrospinal fluid in the brain **Hypoplasia:** incomplete development of an organ or tissue **Kyphosis:** curvature of the spine **Lumbar lordosis:** inward curving of the spine in the lower back region (just above the buttocks) **Malocclusion:** incorrect/imperfect positioning of the teeth when the mouth is closed **Rhizomelia:** shortening of the limbs, with the upper arms and thighs more affected than forearms and calves **Spinal stenosis:** narrowing of the spinal canal housing the spinal cord **Thoracolumbar kyphosis:** curvature of the spine in the mid to lower back

### Medical and developmental considerations

Currently, the clinical management and care of people with achondroplasia vary markedly worldwide, which can lead to variable outcomes for individuals [1]. The International Consensus Statement recommends proactive lifelong and multidisciplinary care of individuals with achondroplasia due to the multisystemic and lifelong nature of the condition. A key aspect of care of individuals with achondroplasia is the multidisciplinary team (MDT). The MDT may include a primary HCP, specialist physicians, and allied health professionals, such as physical therapists, social workers, occupational therapists, psychologists, and genetic counselors. It may be beneficial to have an MDT coordinator act as the primary contact between the family (or adults with achondroplasia) and the MDT. The MDT coordinator should be selected based on their experience with achondroplasia, not their medical specialty, although this role is often filled by a pediatric endocrinologist or genetic disease specialist. Patient advocacy groups or organizations can have an active, complementary role aligned with the work of the MDT. It is through the collaborative work of all these dedicated people that it is possible to offer the best care to individuals with achondroplasia and their families.

### Diagnosis

For individuals with achondroplasia, their families, and HCPs, the critical medical and developmental aspects at the diagnosis stage are confirmation of diagnosis (by genetic testing; Recommendation 5) and early referral to a skeletal dysplasia reference center or clinician experienced in the management of achondroplasia (Recommendation 24).

#### Patient perspective

For couples having a baby with a rare condition, diagnosis can be both confronting and challenging. Regardless of whether the diagnosis is made before or after birth, the communication of the diagnosis by the primary HCP should be careful and allow time both for the information to be presented and for the family to ask questions. Where possible, the HCP should provide information in a variety of formats, as requested by the family; this may be in the form of videos, infographics, and other plain language information sources. Families should be offered psychological support at the time of diagnosis, and informing the family about local (or national) achondroplasia patient advocacy organizations at this time may be beneficial (Recommendation 3).

For couples in which one or both parents have achondroplasia, the approach should be similar, including carefully assessing the need for psychosocial and psychological supports in relation to the life experiences and expectations of the parents. In couples in which both parents have achondroplasia, the probability of homozygous achondroplasia in each of their children is 25%. Homozygous achondroplasia has very low survival; therefore, pre-conception counseling and prenatal testing are recommended (Recommendations 7, 9).

If the diagnosis is prenatal and the couple requests a termination of the pregnancy (if legally allowed in the country or neighboring countries), they should be presented with clear information on the process and offered specific psychological support.

#### Physician perspective

Prenatal genetic testing for achondroplasia is possible for any pregnancy in which achondroplasia is suspected (Recommendation 1); this may be offered if one or both parents have achondroplasia or if features detected on a fetal ultrasound suggest achondroplasia (Recommendation 9). Genetic testing may also be considered to exclude other possible skeletal dysplasias (Recommendations 5–6). As prenatal molecular tools and imaging technologies become more advanced [11], and access to new screening tools increases, it is expected that rare disorders such as achondroplasia will be diagnosed prenatally more frequently.

For a diagnosis made during pregnancy, there may be additional emotional challenges for the parents, and it is recommended that the family meets with specialists experienced in the management of achondroplasia (Recommendation 10). Where possible, this should include members of the MDT that will be supporting the family.

### Infancy (Fig. 1)

Infants with achondroplasia should receive regular follow-up by an MDT, under the guidance of an HCP with experience in achondroplasia (Recommendation 25). If an experienced MDT is not available, the responsible clinician should liaise with an experienced team. During infancy, there should be surveillance for known medical complications of achondroplasia (Recommendations 29–32). Parents will need information and advice about the development of babies with achondroplasia and should be provided with achondroplasia-specific developmental charts (Recommendations 26–27). Children with achondroplasia should receive regular vaccinations following the same national vaccination schedules as their average-height peers (Recommendation 33).

Since the publication of the International Consensus Statement, some pharmacological treatment options for achondroplasia are under clinical development for infants aged less than 2 years [12, 13], one of which has been approved in several countries [14]. The family should be provided with clear, objective information on new research and treatment options for infants, including access and availability in their country. Where applicable, the MDT should present information to families about ongoing clinical trials for infants.

#### Patient perspective

Parents need clear information about the signs of the most complex complications of achondroplasia, including foramen magnum stenosis (Box 3), disordered breathing during sleep, spine issues, and developmental milestones (Fig. 1). Families should be aware that the regular monitoring of infants with achondroplasia involves multiple hospital or clinic visits. These can be time-consuming, and it is important to set up a realistic timetable for appointments and examinations. Where possible, appointments and examinations/evaluations should be aligned to reduce the number of hospital/clinic visits.

**Box 3:** Foramen magnum stenosis and cervicomedullary compression

Impaired bone growth at the base of the skull causes a narrowing of the opening at the base of the skull, called foramen magnum stenosis. The severity of this narrowing differs, and in some infants it may cause compression of the spinal cord at the base of the skull (cervicomedullary compression). Signs of cervicomedullary compression include the following [1]: • Loss or change in motor function or delayed milestones (on achondroplasia-specific charts) • Temporary stoppage of breathing (apnea) • Difficulty swallowing and reduced appetite • Poor weight gain • Abnormal reflexes • Weakness • Involuntary muscle spasms (clonus) In some cases, foramen magnum stenosis may be “silent,” without any of the above signs present, or with very subtle signs. Therefore, infants and children with achondroplasia should receive regular neurological evaluation. MRI is the preferred way to investigate for cervicomedullary compression caused by foramen magnum stenosis. MRI should also be considered for infants without signs in the first months of life to assess foramen magnum size. Surgical intervention (foramen magnum decompression) is recommended in children with signs of cervicomedullary compression and confirming evaluation exams. This should be performed by a surgeon experienced with individuals with achondroplasia. If surgical treatment is required, it should be performed by a neurosurgical team with experience in performing the procedure in infants with achondroplasia – see Box 5. For more details, see Recommendations 34–43, 99, and 116 of the International Consensus Statement [1] MRI: Magnetic resonance imaging	

Families need to be provided with information about safely handling and positioning their baby to protect the neck area and the lower back (Recommendation 28). This needs to be practical advice that includes factors to consider when purchasing safe car seats, strollers/prams, and highchairs.

An experienced HCP should talk with the parents about the typical developmental pathway of babies with achondroplasia. Parents should be reassured that ongoing or intensive physical, speech, and/or occupational therapy may not be required if the infant is developing well according to achondroplasia-specific milestones.

Parents may also require support and advice regarding administrative recognition of their child’s disability, medical insurance implications, and opportunities for governmental support. These will differ according to the healthcare, insurance, and social security structures in each country. Patient advocacy organizations are a valuable resource for supporting parents with these aspects of care.

Family balance is naturally challenged when a new child arrives and is further challenged by the arrival of an infant with achondroplasia. Attention should be given to any siblings, to reinforce family bonds and siblings’ mental health. Attention to siblings should continue throughout life.

#### Physician perspective

It is important for HCPs to inform families that babies up to 2 years old require more frequent surveillance and monitoring to identify related complications early and ensure that development is appropriate. However, surveillance should be individualized, balanced, and coordinated to reduce unnecessary burden on families. The greatest risk of foramen magnum stenosis occurs during infancy, which may lead to cervicomedullary compression (Recommendations 29, 34–43). The International Consensus Statement guideline recommends magnetic resonance imaging (MRI) screening of all infants for this potentially life-threatening complication (Box 3). Routine monitoring during infancy also includes a sleep study (Recommendation 30), hearing evaluations (Recommendation 31), and growth and developmental monitoring using achondroplasia-specific charts (Recommendations 26–27) [2, 15–19]. Information from surveillance and monitoring will help the MDT understand the baby’s individual needs.

Babies with achondroplasia have their own developmental pathway, including delays in gross motor and language skills, when compared with average-statured babies. Ongoing allied health input may not be required if the baby is developing according to achondroplasia-specific developmental milestones.

### Childhood (Fig. 2)

In childhood, there is an ongoing requirement for continued follow-up according to the child’s individual needs. Appointments should review and manage frequently observed complications of achondroplasia, including middle ear effusions, pain and fatigue, sleep issues, spinal curvature (kyphosis), bowed legs (genu varum), dental issues (malocclusion/misalignment of the teeth), and obesity (Recommendations 53–56, 60–63, 65). Psychological and psychosocial support should also be offered to children as required (see section: Psychosocial considerations).

#### Patient perspective

The regular monitoring of children with achondroplasia needs to be tailored to the specific health needs of each child. Healthcare goals and priorities should be continuously assessed, especially as the child matures and is able to participate in their own healthcare decisions. Caregivers and physicians play an important role in supporting the child to understand and accept aspects of care and assessment that may be uncomfortable or unpleasant but are important for their healthcare goals.

The family should be provided with clear information on new research and treatment options. Where applicable, the MDT should present information to families about ongoing clinical trials.

Families should be advised to support participation of their child in appropriate sports and games and encourage an active lifestyle (Recommendation 56). Additional information regarding safe physical activities may be available from patient advocacy groups and/or allied health professionals.

During childhood, it is important to encourage and support independence across all areas, including self-care (Recommendations 57–58). Support for the development of independence skills can be provided by therapists (e.g., physical therapists and occupational therapists) and other clinicians experienced with management of people with achondroplasia. Independence can be assisted by adaptive products. Patient advocacy groups are also a good source of information and assistance.

School integration can be supported in several ways. Some families will feel confident to offer advice and suggestions to schools themselves, while others may prefer support from allied health and/or patient organizations when liaising with schools and advocating for adaptations and aides. Families should be encouraged and supported to use a problem-solving approach and empower children with achondroplasia to look for everyday simple solutions that give them control within different situations. In many cases, “low-tech,” inexpensive adaptations may be appropriate, such as single-step stools in the classroom.

#### Physician perspective

Complications of achondroplasia, such as spinal kyphosis (curvature) and genu varum (bowed legs), may require evaluation by a pediatric orthopedic surgeon and/or pediatric spinal surgeon. Ideally, surgical assessment and interventions should be performed by surgeons with experience in achondroplasia (Recommendations 60–61). If limb-lengthening procedures are being considered, psychological consultation is advised before undertaking such procedures (Recommendation 62).

Another common complication in children is middle ear effusions, which can impact speech and language development (Recommendations 53–54). Speech milestones should be reviewed against achondroplasia-specific charts [19].

The level of pain and fatigue in children is often poorly understood, and it is important that this is considered by families and at medical reviews (Recommendation 63). Questions should address aspects such as variation in pain or tiredness throughout the day, if pain or tiredness reduces mobility at any time, and whether pain or tiredness impacts play, school, or interactions with friends or siblings.

Maintaining healthy activity levels for children with achondroplasia is important. The MDT should support the family to encourage opportunities for the child to try different types of activities (Recommendation 56). Although some types of physical activities can be problematic (e.g., contact sports and gymnastics), many other sports/activities require only slight modifications to allow children with achondroplasia to fully participate and succeed. Families may also need advice on ways to achieve a balance between activity and pain and/or fatigue. For example, using a mobility device over longer distances may reduce fatigue and allow children to participate more in play activities.

Encouragement of healthy eating is also a key component of maintaining a healthy weight for children with achondroplasia. If required, a nutritionist may assist with strategies to support the family and child to control weight.

### Adolescence (Fig. 3)

As is the case during childhood, adolescents with achondroplasia require individualized follow-up plans (Recommendation 67). The presence and management of pain and the emotional and mental health effects of pain should be carefully monitored during adolescence (Recommendation 68). Routine follow-up should also include discussion of potential adaptive equipment use and/or environmental modifications at home and school that maximize independence (Recommendation 70).

As with younger children, adolescents may be at risk of obesity and should be provided with education regarding healthy eating and encouraged to foster an active lifestyle (Recommendations 71–72). Mental health support should be offered as needed during this life stage, including professional support to assist with strategies for school, employment, and social environments (Recommendation 73). Some adolescents may need specific support to accept and use their adaptive equipment [1].

#### Patient perspective

Adolescents with achondroplasia may need support to understand that their condition is lifelong. They should be made aware of the more frequent complications of achondroplasia in adulthood and the early signs, particularly for spinal stenosis. During later adolescence, it is important to set specific appointments for transition to an adult clinic (if available) or MDT (Recommendation 131). Adolescents should be empowered to identify signs of complications and to learn how to manage their own health care, including being supported to ensure that their medical follow-up is tailored to their individual needs.

#### Physician perspective

Routine monitoring continues during adolescence. If there are symptoms of spinal stenosis, the adolescent should be referred to a spinal service with experience in achondroplasia (Recommendation 69). Typical symptoms include ongoing back pain; radiating pain, tingling, or numbness in the arms or legs; pain while walking or standing; reduced walking distance; or bladder or bowel dysfunction (Recommendation 74; Box 4). In addition to routine screening and monitoring, adolescents should be offered the opportunity to discuss achondroplasia inheritance patterns and related issues with HCPs; this may be with a genetic counselor, family counselor, and/or psychologist (Recommendation 7). The MDT should be proactive in assisting with transition of the adolescent to adult healthcare environments.

**Box 4:** Symptomatic spinal stenosis

Symptomatic spinal stenosis may develop in people with achondroplasia because the diameter of the spinal canal (housing the spinal cord) is reduced, resulting in compression of the spinal cord. A suite of symptoms may develop because of the pressure on the spinal cord. Signs of spinal compression include the following [4]: • Pain and/or radiating pain into the legs or buttocks • Reduced sensations in the legs and feet • Reduced muscle strength in the legs and feet • Urinary incontinence • Bowel incontinence Symptomatic spinal stenosis is a key complication of achondroplasia that requires accurate diagnosis and careful management. It typically develops in adolescence or adulthood, and symptoms may progress rapidly. A full spinal MRI is recommended for patients exhibiting the symptoms listed above If MRI imaging indicates spinal cord compression, a referral to a spine center experienced in spinal stenosis management of patients with achondroplasia is recommended [1]. Management may include weight loss, physical therapy, and/or surgical decompression. For more details, see Recommendations 69, 74–75, 85, 89, and 120 of the International Consensus Statement [1] MRI: magnetic resonance imaging	

### Adulthood (Fig. 4)

Achondroplasia-related medical complications may occur during any life stage. Individuals should be informed about the signs and symptoms of the most frequent medical complications in adulthood. Monitoring during adulthood should include consideration of symptoms of spinal stenosis, pain, bladder/bowel problems, sleep apnea, blood pressure, hearing loss, mental health, and any routine health checks advised for the general community (Recommendations 74, 76–80, 82). People with achondroplasia may require ongoing support in their community, work, and/or social environments, including assistive devices and car adaptations, to maximize their independence (Recommendation 84).

#### Patient perspective

The medical needs of adults with achondroplasia will differ between individuals. Pain such as leg or back pain can be common during adulthood and may increase with age. Thus, adults may need support to monitor and control pain, including consideration of the effect of pain on their mood, function, and social life (Recommendations 79, 118). Adults experiencing chronic pain are best managed using a multidisciplinary approach (Recommendation 119).

Adults with achondroplasia should be supported to maintain their general physical and mental health, including development of healthy nutrition and exercise habits (Recommendation 125) and psychological support whenever needed or requested (Recommendations 82, 129).

Individuals with achondroplasia who wish to become parents should be provided with access to genetic counseling and pre-conception health evaluation (Recommendations 7–11, 81).

#### Physician perspective

Adults with achondroplasia should be encouraged to attend clinics where their condition is understood. Where no adult achondroplasia-specific clinics exist, adults should be encouraged to develop connections with local clinicians to create their MDT. The individual and their MDT should work together to anticipate and manage medical complications.

Early detection and proper management of spinal stenosis are important, including prompt referral to a spine center and clinicians with expertise in achondroplasia (Recommendations 74–75). If spinal surgery is indicated, this should be performed by a surgical team with experience with patients with achondroplasia (Recommendation 121; Box 5).

**Box 5:** Surgery and anesthesia

Some complications associated with achondroplasia may need surgical intervention or management. Common surgeries include placement of ear tubes to prevent conductive hearing loss in children with recurrent otitis media, adenotonsillectomy (often performed in children to open airways to reduce obstructive sleep apnea), and spinal and orthopedic surgeries. If surgery is being considered, ideally it should be performed by a surgical team familiar with achondroplasia (where available). Decisions for operations are best made by surgeons working in conjunction with the achondroplasia MDT [1]. Anesthesia can be challenging in patients with achondroplasia, especially in older patients. Challenges include difficult intravenous access [20] and difficulties with bag-mask ventilation due to small mouth opening, narrow nostrils, midface hypoplasia, and a short neck with limited head/neck movement [21, 22]. Endotracheal tubes smaller than the standard sizes may be needed due to smaller airway dimensions [21]. The International Consensus Statement recommends the following for general anesthesia in individuals with achondroplasia [1]: • Anesthesia should take place preferentially in hospitals with HCPs experienced in caring for patients with achondroplasia. • Head and neck movement should be avoided/minimized during bag-mask ventilation due to foramen magnum stenosis. Airway adjuncts for difficult bag-mask ventilation should be readily available, and video laryngoscopes should be used to avoid neck movement. • Thorough pre-anesthesia assessment is required for people with achondroplasia. Pre-assessment should include airway assessment, range of neck movement, and history of snoring/sleep-disordered breathing [22]. These should also be used to guide the patient’s post-anesthetic stay in the hospital. For anesthesia in obstetric care, it is recommended that there is early discussion regarding mode of birth and anesthesia options [1]. For more details, see Recommendations 16, 83, 114–117, and 121 of the International Consensus Statement [1] HCP: Healthcare professional; MDT: Multidisciplinary team	

Pain prevalence is often high in adults with achondroplasia [4, 23, 24] and should be monitored regularly (Recommendations 79, 118). Pain can be related to spinal stenosis [4] and/or spine deformity and can be influenced by weight [25]. Fatigue can also be a problem but has not been studied in detail. Adults need to be empowered to discuss pain and fatigue with their doctors to look at practical ways to support management of these issues. Patient-reported outcome scales, such as the Brief Pain Scale [26] and the Screening Tool for Everyday Mobility and Symptoms [27], can be used to monitor changes over time.

Some medical complications, such as hearing issues, bladder and bowel problems, and sleep disorders, may not be recognized as related to their condition by adults with achondroplasia but can significantly impact quality of life. Bladder and bowel issues may be uncomfortable topics for many people, but because these can be related to spinal stenosis, it is important that such problems are discussed with HCPs. Clinicians need to encourage adults to self-advocate and ask questions about the possible need to investigate these complications.

Due to an increased need for cesarean section and the potential for respiratory complications, women with achondroplasia who become pregnant will require specific care by an obstetrician (Recommendation 12). Prenatal care should include prenatal anesthetic assessment; surveillance for respiratory, cardiac, or skeletal complications; and fetal ultrasounds (Recommendations 13–15).

For adults with achondroplasia, independence at home, at work, and in the community is a key aspect in life. Allied health input can support independence with activities of daily living by providing aids and adaptations that continue to enable independence and participation in employment and leisure activities (Recommendations 122–124). Mobility devices, adaptive equipment, and environmental modifications can maximize independence, and these should be considered in accordance with individual needs (Recommendation 124).

### Psychosocial considerations

Psychosocial considerations should be included in best practice clinical care and management of people with achondroplasia. The International Consensus Statement provides several recommendations for psychosocial issues and family support (Recommendations 128–136) and highlights the importance of social resources, particularly for young people with achondroplasia [1]. The International Consensus Statement also addresses psychological and psychosocial needs at each stage of life, and the need for psychosocial support to be a routine part of health care [1].

#### Patient perspective

Having a child with achondroplasia can be life-changing when the diagnosis is unexpected. At the time of diagnosis, and throughout infancy, it is important to consider parents’ needs and identify support options for them. When providing information about achondroplasia and possible medical complications, this should be appropriate for both the age and developmental stage of the child and balanced against the emotional needs of the parents. Information about potential medical complications should also be provided within the context that people with achondroplasia can live happy, healthy, and productive lives. The family may need psychosocial support, ideally from a network that includes social workers, psychologists, patient advocacy organizations, teachers, extended family, and friends. These networks can provide practical, informational, educational, emotional, and psychological support as needed by the family.

As the child grows, the needs of the family and the child will change, and the support structure may need to be adapted. The emotional and psychological support needs of the individual with achondroplasia and their family should be reassessed as required. In particular, children and adolescents may benefit from quality-of-life assessment with specific tools to identify whether their needs are being met. The need for more formal mental health support should be considered for children and adolescents as part of their regular health care (Recommendations 130, 135).

Families should be referred to, and encouraged to reach out to, a patient advocacy organization dedicated to achondroplasia to receive information and maximize their psychosocial supports. Where there is no local or national achondroplasia-specific organization, families can be referred to a rare disease organization or international support group.

Having a rare condition that is also characterized by specific physical features that are very different from the average population may raise diverse social challenges, and it is important to foster positive emotional resilience and a socially active and engaged life. Particularly during school years, attention should be paid to the possibility of bullying and social exclusion. The promotion of social acceptance and inclusion of people with achondroplasia is a joint endeavor of the individual’s family, patient advocacy groups, and HCPs (Recommendation 136).

The MDT should support the family to foster social experiences appropriate to the child’s development (e.g., baby swim classes may be beneficial for infants and their parents, and extracurricular activities, including sports activities, may be beneficial for older children and adolescents).

#### Physician perspective

The role of the MDT should be to optimize the physical and psychosocial well-being of the individual with achondroplasia (Recommendations 134–136). The goal is that individuals with achondroplasia are empowered to fully participate in society. It is also important that joint collaborations between HCPs, patient advocacy groups, and families work to promote the social awareness of achondroplasia and acceptance of physical differences across society (Recommendation 136).

Psychosocial support may be required at multiple points throughout the patient’s life span. The MDT is well placed to coordinate and recommend this psychosocial support (Recommendation 135). Facilitating contact with patient advocacy organizations can provide important psychosocial and practical support for families. Having people who know and understand the condition is important to help the family take in and process information, particularly at the early post-diagnosis stage.

The MDT can also provide direct psychosocial support. During infancy and childhood, families require clear information about existing resources and help in deciding which therapies may be necessary. The MDT can also support families to accept their child’s growth and development. Although families need to be aware of signs of potentially serious complications, the foremost role of the parents is to enjoy their child and their lives together as a family, and they should be supported to achieve this goal.

## The healthcare system

### Patient perspective

Healthcare systems should support lifelong, multidisciplinary care for individuals with achondroplasia and their families (Recommendation 135). Because achondroplasia requires an individualized approach in a multidisciplinary clinical setting, healthcare systems must be adaptable and recognize that no single management strategy will apply to all people with achondroplasia. Many individuals with achondroplasia may experience few complications and interventions throughout their lives, whereas others may require several or multiple achondroplasia-related medical challenges. Therefore, the healthcare system should support the MDT to prioritize understanding the individual’s current health state and individual risks for major complications of the condition. Overall, empowering individuals and families to be proactive regarding their priorities and choices should be integral/central to developing the most appropriate management plan.

Specialist treatment centers for achondroplasia are not available or accessible in all countries. Thus, it is crucial that physicians who are less experienced in the follow-up and care of patients with achondroplasia are made aware of the International Consensus Statement recommendations [1] and achondroplasia-specific developmental charts [2, 15–18, 27]. Less experienced HCPs should refer to or discuss with experts when required. Patient advocacy organizations play a role in raising awareness of achondroplasia among HCPs, promoting treatment guidelines and growth charts, and empowering individuals with achondroplasia to proactively manage their health care.

### Physician perspective

The physician’s goal should be to deliver the best care possible, regardless of country of residence. Where possible, individuals with achondroplasia should receive multidisciplinary medical care that includes access to social and psychological support (Recommendation 135). Care should be taken, in alignment with clinical needs, to avoid over-servicing people with achondroplasia. This is particularly important for infants and children who are developing well according to achondroplasia-specific milestones. If specific resources are not available (e.g., MRI in less developed countries), we encourage HCPs to contact experts outside of their country for guidance beyond this document.

Although multidisciplinary care is as important for adults with achondroplasia as it is for children, expertise and services for multidisciplinary care of adults are often limited [28]. There is a great need to advocate for adult services for achondroplasia and to foster physicians’ interest in this care area.

A list of the specialty areas that are most often required for treatment and care of people with achondroplasia is provided in Box 6.

**Box 6:** List of specialty areas important for care of individuals with achondroplasia, including recommendation numbers from the International Consensus Statement

1. Spinal care (Recommendations 85–89) 2. Care of lower limbs (Recommendations 90–97) 3. Respiratory care, including sleep-disordered breathing (Recommendations 98–105) 4. Ear, nose, and throat (Recommendations 106–111) 5. Orthodontics and maxillofacial surgery (Recommendations 112–114) 6. Anesthesia (Recommendations 115–117) 7. Pain and function (Recommendations 118–124) 8. Nutrition, physical activity, and weight management (Recommendations 125–127) 9. Psychosocial issues and support (Recommendations 128–136) For more details, see the International Consensus Statement [1].	

## Conclusions

Most people with achondroplasia live fully independent, productive, and socially engaged lives, although they may experience medical, functional, and psychosocial challenges at various times. The goal for lifelong care of individuals with achondroplasia is to optimize their physical and mental health through provision of individualized care and to promote participation and inclusion in society. This requires surrounding them with a knowledgeable and supportive family, a well-informed team of medical professionals, and suitable psychosocial supports. Making early connections with a local and/or national support group or advocacy organization may facilitate acceptance of the diagnosis and can be a strong pillar of the psychosocial support structure for individuals with achondroplasia. Support groups/advocacy organizations provide valuable information, support, and social connections with other individuals with achondroplasia and their families.

## Electronic supplementary material

Below is the link to the electronic supplementary material.

Supplementary material 1

## Data Availability

No datasets were generated or analysed during the current study.

## Abbreviations

HCP: Healthcare professional
MDT: Multidisciplinary team
MRI: Magnetic resonance imaging
