# Supplementary material for: An overview of the International Consensus Statement on achondroplasia
Source: Orphanet J Rare Dis. 2026 Jan 20;21:34. doi: 10.1186/s13023-025-04189-y (PMC12857165; doi:10.1186/s13023-025-04189-y)
Supplement: Supplementary file 1 — Supplementary material 1 [file 13023_2025_4189_MOESM1_ESM.docx]

Supplementary Information

TITLE

An overview of the International Consensus Statement on achondroplasia

AUTHORS

Inês Alves^1,2^

Svein Otto Fredwall^3^

Michael Hughes^4^

Penelope J. Ireland^5,6^

Morrys C. Kaisermann^7,8^

Ravi Savarirayan^9^

AUTHORS’ AFFILIATIONS

^1^ANDO Portugal, Évora, Portugal

^2^Comprehensive Health Research Centre (CHRC), Health and Sport Department, School of Health and Human Development, Évora University, Évora, Portugal

^3^ National Centre for Rare Diseases, Sunnaas, Nesodden, Norway

^4^Biotech Industry Liaison Committee Chair, Little People of America, Sonoma, CA, USA

^5^School of Health and Rehabilitation Sciences, University of Queensland, Brisbane, Queensland, Australia

^6^Queensland Children’s Hospital, Brisbane, Queensland, Australia

^7^Growing Stronger, Saratoga, CA, USA

^8^Fundación ALPE Acondroplasia, Gijón, Spain

^9^Murdoch Children’s Research Institute, Royal Children’s Hospital, University of Melbourne, Parkville, Victoria, Australia

*CORRESPONDENCE

Penelope J. Ireland

School of Health and Rehabilitation Sciences, University of Queensland, Brisbane, Queensland, Australia

Tel: +61-7-3068 2950

Email: [penny.ireland@health.qld.gov.au](mailto:penny.ireland@health.qld.gov.au)

**LIST OF SUPPLEMENTARY information**

**Additional file 1** Patient advocacy organizations around the world

**Additional file 2** Growth and development in children with achondroplasia

**Additional file 3** Positioning and handling infants and young children with achondroplasia

**Additional file 4** Guidance for weight control in children and adults with achondroplasia

Additional file 1 Patient advocacy organizations around the world

| Region | Country | Organization (hyperlink) |
| --- | --- | --- |
| Africa | Guinea | [Club Le Fromager, Personnes de Petite Taille - CFPPT (Facebook)](https://www.facebook.com/Club-Le-Fromager-Personnes-de-Petite-Taille-116844115330612/) |
|  | Kenya | [Short Stature Society of Kenya - SSSK (Facebook)](https://www.facebook.com/Short-Stature-Society-of-Kenya-SSSK-799821503449817/)  [Little People of Africa Foundation (Facebook)](https://www.facebook.com/littlepeopleafrica/) |
|  | South Africa | [Little People of South Africa (Facebook)](https://www.facebook.com/LittlePeopleOfSouthAfrica/) |
|  | Uganda | [Little People of Uganda (Facebook)](https://www.facebook.com/littlepeopleinuganda/) |
| Asia and Middle East | India | [The Little People of India (Facebook)](https://www.facebook.com/littlepeopleindia/) |
|  | Iraq | [Short Statured People of Iraq (Facebook)](https://www.facebook.com/sspiraq/) |
|  | Japan | [Glory to Achondroplasia - GTA](http://glory-to-achondroplasia.com/) |
|  | Malaysia | [Pertubuhan Kebangsaan Orang Kerdil Malaysia - PKOKM (National Organization of Malaysian Dwarfs) (Facebook)](https://www.facebook.com/orangkerdilmalaysia/) |
|  | Pakistan | [Little People of Pakistan (webpage)](http://lppakistan.blogspot.pt/)  [Little People of Pakistan (Facebook)](https://www.facebook.com/p/Little-People-of-Pakistan-100067145654100/) |
|  | Philippines | [Big Dreams for Little People - Philippines Inc. (Facebook)](https://www.facebook.com/BDLP.Phil2017) |
| Europe | Austria | [Bundesverband Kleinwüchsiger Menschen und ihrer Familien – BKMF (Federal Association of Little People and Their Families) (webpage)](http://www.bkmf.at/)  [BKMF (Facebook)](https://www.facebook.com/bkmf.oesterreich/) |
|  | Bulgaria | [Little People of Bulgaria](http://www.lpbulgaria.org/)  [Little People of Bulgaria (Facebook)](https://www.facebook.com/lpbulgaria.org/) |
|  | Czechia | [Paleček (webpage)](http://www.ospalecek.cz/)  [Paleček (Facebook)](https://www.facebook.com/www.ospalecek.cz/) |
|  | Denmark | [Dværgeforeningen - DVF (Danish Dwarf Association)](http://lfvdk.dk/) |
|  | Finland | [Lyhytkasvuiset-Kortväxta ry (webpage)](http://www.lyhytkasvuiset.fi/)  [Lyhytkasvuiset-Kortväxta ry (Facebook)](https://www.facebook.com/lyhytkasvuiset/) |
|  | France | [Association des Personnes de Petite Taille - APPT (Association of People of Small Size) (Facebook)](https://www.facebook.com/assoappt/) |
|  | Germany | [Bundesverband Kleinwüchsige Menschen und ihre Familien e.V – BKMF (German Association for People of Short Stature) (webpage)](http://www.bkmf.de/)  [BKMF (Facebook)](https://www.facebook.com/BKMFeV/)  [BundesselbsthilfeVerband Kleinwüchsiger Menschen e.V. - VKM (Federal Association for the Promotion of People of Short Stature) (webpage)](http://www.kleinwuchs.de/)  [VKM (Facebook)](https://www.facebook.com/groups/354957907965348/) |
|  | Hungary | [Kisemberek Társasága (Little People of Hungary) (webpage)](http://www.kisemberek.hu/)  [Little People of Hungary (Facebook)](https://www.facebook.com/DavidKisemberekTarsasaga) |
|  | Ireland | [Little People of Ireland (Facebook)](https://www.facebook.com/pages/Little-People-of-Ireland/124797444227496)  [Dwarf Sports Association of Ireland - DSAI (webpage)](http://www.dsairl.ie/)  [DSAI (Facebook)](https://www.facebook.com/dsairl/) |
|  | Italy | [AISAC Onlus](http://aisac.it/) |
|  | Kosovo | [Little People of Kosovo](http://www.lpokosova.com/) |
|  | Netherlands | [Belangenvereniging Van Kleine Mensen (BVKM)](http://www.bvkm.nl/)  [BVKM (Facebook)](https://www.facebook.com/groups/bvkm.nl/) |
|  | Norway | [Norsk Interesseforening for Kortvokste - NiK (Little People of Norway) (webpage)](http://www.kortvokste.no/)  [NiK (Facebook)](https://www.facebook.com/kortvokste/) |
|  | Poland | [Klub Nieduzi (Little People of Poland - LPP) (webpage)](http://www.nieduzi.org/)  [LPP (Facebook)](https://www.facebook.com/nieduzi) |
|  | Portugal | [ANDO Portugal - Associação Nacional de Displasias Ósseas (National Association for Skeletal Dysplasias) (webpage)](http://www.andoportugal.org/)  [ANDO Portugal (Facebook)](https://www.facebook.com/andoportugal/) |
|  | Romania | [Asociatia Oamenilor Mici (Association of Little People Romania)](http://www.asociatiaoamenilormici.com/)  [Asociatis Oamenilor Mici (Facebook)](https://www.facebook.com/AsociatiaOamenilorMici/) |
|  | Russia | [маленькие люди россии - MлP (Little People of Russia - LPR)](https://lp-ru.ru/) |
|  | Serbia | [Ahondroplazija Srbija (Little People of Serbia) (Facebook)](https://www.facebook.com/littlepeopleofserbia/) |
|  | Slovakia | [Palčekovia (Little People of Slovakia)](http://www.palcekovia.sk/) |
|  | Slovenia | [Drustvo Malih Ljudi Slovenije (Little People of Slovenia) (email)](mailto:mls@siol.net) |
|  | Spain | [AFAPAC - Asociación de Familiares y Afectados de Patologías del Crecimiento (webpage)](http://afapac.org/)  [AFAPAC (Facebook)](https://www.facebook.com/Afapac-1692040517719642/)  [Asociación Nacional Para Problemos de Crecimeinto - CRECER](http://www.crecimiento.org/)  [Fundación Alpe Acondroplasia (webpage)](http://www.fundacionalpe.org/en/)  [Fundación Alpe (Facebook)](https://www.facebook.com/FundacionALPE/)  [Sports PTB (Facebook)](https://www.facebook.com/sports.ptb) |
|  | Sweden | [Föreningen för Kortvuxna DHR - FKV (Association for People of Short-Statures in Sweden - APSS) (webpage)](http://www.fkv.se/)  [FKV (Facebook)](https://www.facebook.com/groups/212592527919/) |
|  | Switzerland | [Verein Kleinwüchsiger Menschen - VKM (Association of Little People)](http://www.kleinwuchs.ch/)  [Association Romande des Personnes de Petite Taille - ARRPT](http://www.arppt.ch/) |
|  | United Kingdom | [Dwarf Sports Association UK - DSAuk (webpage)](http://www.dsauk.org/)  [DSAuk (Facebook)](https://www.facebook.com/DSAUK/)  [FEST - Foundation Exploring Skeletal Dysplasia Together (webpage)](https://www.myskeletaldysplasia.org.uk/)  [FEST (Facebook)](https://www.facebook.com/MySkeletalDysplasia)  [Little People UK - LPUK (webpage)](http://www.littlepeopleuk.org/)  [LPUK (Facebook)](https://www.facebook.com/Little-People-UK-314441205267611/)  [Restricted Growth Association - RGA (webpage)](http://www.restrictedgrowth.co.uk/)  [RGA (Facebook)](https://www.facebook.com/RGAUK/)  [Short Stature Scotland (Facebook)](https://www.facebook.com/groups/240028636037894/) |
| North America | Canada | [Association of Little People of Alberta - ALPA](http://www.albertalittlepeople.com/)  [ALPA (Facebook)](https://www.facebook.com/groups/137433529222/)  [Association Québécoise des Personnes de Petite Taille - AQPPT (Quebec Association of Little People)](http://www.aqppt.org/)  [AQPTT (Facebook)](https://www.facebook.com/AQPPT/?locale=fr_FR)  [Dwarf Athletic Association of Canada - DAAC](http://daaca.ca/)  [DAAC (Facebook)](https://www.facebook.com/groups/DwarfAthleticsCanada/)  [Little People of British Columbia – LPBC](https://littlepeopleofbc.org/)  [LPBC (Facebook)](https://www.facebook.com/LPBC.collective/)  [Little People of Canada - LPC](https://littlepeopleofcanada.com)  [LPC (Facebook)](https://www.facebook.com/LittlePeopleCan)  [Little People of Manitoba - LPM](https://littlepeoplemanitoba.wordpress.com/)  [LPM (Facebook)](https://www.facebook.com/LPManitoba/)  [Little People of Ontario - LPO](http://www.lpo.on.ca)  [LPO (Facebook)](https://www.facebook.com/groups/littlepeopleofontario/) |
|  | Mexico | [Consejo Nacional Gente Pequeña Mexico - CNGP (National Council for Little People Mexico) (Facebook)](https://www.facebook.com/CNGPMexico/) |
|  | United States | [Dwarf Athletic Association of America - DAAA](http://www.daaa.org/)  [DAAA (Facebook)](https://www.facebook.com/DwarfAthleticAssociationOfAmerica/)  [Growing Stronger](http://www.growingstronger.org/)  [Growing Stronger (Facebook)](https://www.facebook.com/TeamGrowingStronger/)  [Human Growth Foundation - HGF](http://hgfound.org/)  [HGF (Facebook)](https://www.facebook.com/hgf.growing.together/)  [Little People of America (LPA)](https://www.lpaonline.org/)  [LPA (Facebook)](https://www.facebook.com/LittlePeopleofAmerica)  [The MAGIC Foundation](https://www.magicfoundation.org/)  [The MAGIC Foundation (Facebook)](https://www.facebook.com/TheMAGICFoundation/)  [Understanding Dwarfism](http://www.udprogram.com/)  [Understanding Dwarfism (Facebook)](https://www.facebook.com/understandingdwarfismprogram) |
| Oceania | Australia | [Short Statured People of Australia (SSPA)](https://www.sspa.org.au/) |
|  | New Zealand | [Little People of New Zealand](http://www.lpnz.org.nz/)  [Little People of New Zealand (Facebook)](https://www.facebook.com/littlepeoplenz/) |
| South and Central America | Argentina | [Achondroplasia Argentina - ACONAR (webpage)](https://aconar.org.ar/)  [ACONAR (Facebook)](https://www.facebook.com/acondroplasia.argentina) |
|  | Bolivia | [Asociación Personas Talla Baja Bolívia (Bolivian Association for People With Dwarfism) (Facebook)](https://www.facebook.com/groups/926661140761389) |
|  | Brazil | [Associação Nanismo Brasil - ANNABRA (Facebook)](https://www.facebook.com/annabrananismo/)  [Associação de Nanismo do Estado do Rio de Janeiro - ANAERJ (Rio de Janeiro State Dwarfism Association) (Facebook)](http://www.facebook.com/anaerj)  [Instituto Nacional de Nanismo - INN (webpage)](https://institutonacionaldenanismo.com.br/)  [INN (Facebook)](https://www.facebook.com/nanismobr)  [Somos Todos Gigantes (We Are All Giants) (Facebook)](https://www.facebook.com/somostodosgigantes/) |
|  | Chile | [Asociación Padres Acondroplasia Chile (webpage)](https://www.acondro.cl/)  [Asociación Padres Acondroplasia Chile (Facebook)](https://www.facebook.com/Acondro.cl) |
|  | Colombia | [Corporacion Pequeñas Personas Latinas (Facebook)](https://www.facebook.com/groups/CPPLMEDELLIN/about)  [Red de redes latinas de pequeños y org de peq yPersonas (Facebook)](https://www.facebook.com/groups/pequenaspersonaslatinas/about)  [Asociación Pequeños Gigantes (webpage)](https://asociacion-pequenos-gigantes-de-colombia.webnode.com.co/)  [Asociación Pequeños Gigantes (Facebook)](https://www.facebook.com/profile.php?id=100064817952102) |
|  | Costa Rica | [Asociacion Pro-Gente Pequeña de Costa Rica - APGPCR (Association Pro Short Statured People of Costa Rica) (Facebook)](https://www.facebook.com/APGPCR) |
|  | Dominican Republic | [Personas Pequeñas de la República Dominicana](https://www.facebook.com/personaspequenasrd/) |
|  | Ecuador | [Asociación Ecuatoriana de Personas de Talla Baja - AEPTB (Ecuatorian Dwarfism Association) (Facebook)](https://www.facebook.com/AEPTB/) |
|  | Uruguay | [Asociación Acondroplasia Uruguay (Uruguayan Achondroplasia Association) (Facebook)](https://www.facebook.com/acondroplasia.uruguay/) |

Additional file 2 Growth and development in children with achondroplasia

How to measure infants and children with achondroplasia (adapted from [1])

When measuring children, cooperation with the child is needed to obtain reliable and reproducible results. Ideally, the measurement reading should not be taken until the desired measurement position is obtained. Below we briefly describe some measurement techniques. More detailed instructions, along with illustrations, are available in reference [2].

***Length and height***

Before a child can stand, measure length with the child lying on their back on a measuring board. For best results, two people are needed to secure both heels to the bottom plate of the measuring board.

Once a child can stand, height can be measured standing. If the child has a significant curve in the lower back, *gentle* external pressure can be applied to align the back to the measuring support area. However, if this technique is used, then all subsequent measurements should use this technique.

Height values may decrease between morning and afternoon for all children due to compression of spinal disks. If measuring regularly, the time of day of measurement should be standardized where possible.

***Weight, waist circumference, and body mass index (BMI)***

Weight should be measured with the child undressed or in light clothing, using a calibrated scale. Waist circumference is measured midway between the last rib and the iliac (hip) crest.

BMI is calculated from weight and height (BMI = weight [kg] / height [m]^2^) and therefore can amplify measuring inaccuracies. It can appear more irregular when plotted on a graph than height or weight alone. BMI may be misleading for evaluation of metabolic health in individuals with achondroplasia [2,3].

***Head circumference***

Head size is measured as the maximum circumference. A non-stretchable tape measure should be used.

Growth and development charts for children with achondroplasia

Several public domain growth and development charts are available. We have provided links below to several options.

***Beyondachondroplasia.org [4–6]***

- Height: [https://www.beyondachondroplasia.org/height-for-age](https://www.beyondachondroplasia.org/en/?option=com_content&view=article&id=11&Itemid=274)
- Weight: [https://www.beyondachondroplasia.org/en/weight-for-age](https://www.beyondachondroplasia.org/en/?option=com_content&view=article&id=58&Itemid=274)
- BMI: [https://www.beyondachondroplasia.org/en/BMI](https://www.beyondachondroplasia.org/en/?option=com_content&view=article&id=60&Itemid=274)
- Head circumference: [https://www.beyondachondroplasia.org/en/head-circumference](https://www.beyondachondroplasia.org/en/?option=com_content&view=article&id=59&Itemid=274)

***Achondroplasia-growthcharts.com [3, 7, 8]***

- Height: <https://www.achondroplasia-growthcharts.com/height-development/>
- Weight and BMI: <https://www.achondroplasia-growthcharts.com/weight-and-bmi/>
- Head circumference: <https://www.achondroplasia-growthcharts.com/head-circumference/>

References

1. Neumeyer L, Merker A, Hagenäs L. Recommended measuring techniques. <https://www.achondroplasia-growthcharts.com/measurement-techniques/>. Accessed 18 Nov 2025.
2. Hoover-Fong J, Semler O, Barron B, Collett-Solberg PF, Fung E, Irving M, et al. Considerations for Anthropometry Specific to People with Disproportionate Short Stature. Adv Ther. 2025;42:1291–311.
3. Merker A, Neumeyer L, Hertel NT, et al. Growth in achondroplasia: development of height, weight, head circumference, and body mass index in a European cohort. Am J Med Genet A. 2018;176:1723–34.
4. del Pino M, Fano V, Lejarraga H. Growth references for height, weight, and head circumference for Argentine children with achondroplasia. Eur J Pediatr. 2011;170:453–9.
5. Hoover-Fong J, McGready J, Schulze K, Alade AY, Scott CI. A height-for-age growth reference for children with achondroplasia: expanded applications and comparison with original reference data. Am J Med Genet A. 2017;173:1226–30.
6. Tofts L, Das S, Collins F, Burton KLO. Growth charts for Australian children with achondroplasia. Am J Med Genet A. 2017;173:2189–200.
7. Merker A, Neumeyer L, Hertel NT, Grigelioniene G, Mohnike K, Hagenäs L. Development of body proportions in achondroplasia: sitting height, leg length, arm span, and foot length. Am J Med Genet A. 2018;176:1819–29.
8. Neumeyer L, Merker A, Hagenäs L. Clinical charts for surveillance of growth and body proportion development in achondroplasia and examples of their use. Am J Med Genet A. 2020;185:401–12.

Additional file 3 Positioning and handling infants and young children with achondroplasia

Babies and young children with achondroplasia have relatively large heads and loose joints. The looseness of control of the neck is a particular area of concern. There are three major considerations regarding position of an infant/young child with achondroplasia [1]:

- Breathing: the chest volume is relatively small, and the windpipe is soft and compressible, so a bent-over position with the chin toward the chest may restrict the windpipe and cause breathing difficulty.
- Spinal protection: the head and neck need to be supported to reduce the risk of spinal cord complications.
- Back support: many babies with achondroplasia have a flexible back and thoracolumbar kyphosis. Back support reduces the risk of progressive, fixed, thoracolumbar kyphosis.

Positioning guidelines [1, 2]

- **Avoid trying to get the baby to sit** until the child tries to do it themselves.
- **No** **unsupported sitting** for the first year; no propping up to sit with pillows, etc.
- When not sleeping in a crib/cot or bed, the safest place for an infant/child with achondroplasia is **on the floor**. Babies can be positioned **on their back, side, or stomach**.

Handling positions for babies with achondroplasia [1, 2]

- **Support** **the baby’s head and neck** at all times.
- When **lifting** the baby, use one hand to support the head and neck, and the other to support the lower back.
- When **carrying** the baby, keep them fully reclined, with head, neck, and lower back supported.

Car seats, strollers/prams, and supportive devices [1, 2]

- **Avoid soft devices** that bend the back, including soft infant carriers, umbrella strollers, and soft infant swings.
- **Use hard-backed baby carriages**, strollers/prams, and supportive devices.
- **Avoid infant walkers, bouncers, and “jumper” devices**. These devices force head support when the child may not be ready and can cause neck injury.
- **Use a rear-facing car seat until the child is 3–4 years old** (or for as long as the child needs extra neck support).
- **Use padding in the infant car seat** around the baby’s head for support to prevent the chin-on-chest position.

References

1. Reid CS. Handling the newborn and young infant with achondroplasia [information sheet]. [https://lpamrs.memberclicks.net/assets/documents/Handling%20the%20Newborn%20with%20Achon[1].doc](https://lpamrs.memberclicks.net/assets/documents/Handling%20the%20Newborn%20with%20Achon%5b1%5d.doc). Accessed 18 Nov 2025.
2. Alves I. Basic indications to handle a baby/young child with achondroplasia. <https://www.beyondachondroplasia.org/en/library/guidelines/152-basic-indications-to-handle-a-baby-young-child-with-achondroplasia>. Accessed 18 Nov 2025.

Additional file 4 Guidance for weight control in children and adults with achondroplasia

Obesity is a major health problem for many people with achondroplasia, and it requires complex clinical management. Excessive weight may exacerbate complications such as sleep apnea and spinal stenosis [1–3].

Obesity in achondroplasia may develop atypically and early, such as abdominal obesity in childhood [2]. At present, the underlying metabolic pathways that may contribute to obesity in achondroplasia are not well understood, but there is increasing research interest in this area.

There are several recommendations for medical management of obesity in patients with achondroplasia based on current evidence [1–4]. We have summarized these below.

- Regular individual assessments of nutrition and dietary guidance are recommended for people with achondroplasia [1, 3].
- It is best to achieve dietary changes gradually, considering a person’s habits, food preferences, bodily sensations (hunger/satiety), and satisfaction with food [2].
- Psychological support may be helpful to facilitate changes in diet and physical activity [2].
- There are limited data available for bariatric surgery in individuals with achondroplasia [4]. The risks associated with surgery and anesthesia should be considered if bariatric surgery is being contemplated.
- Regular physical activity is recommended for all people with achondroplasia [1].

References

1. Savarirayan R, Ireland P, Irving M, Thompson D, Alves I, Baratela WAR, Betts J, et al. International Consensus Statement on the diagnosis, multidisciplinary management and lifelong care of individuals with achondroplasia. Nat Rev Endocrinol. 2022;18:173–89.
2. Saint-Laurent C, Garde-Etayo L, Gouze E. Obesity in achondroplasia patients: from evidence to medical monitoring. Orphanet J Rare Dis. 2019;14:253.
3. Madsen A, Fredwall SO, Maanum G, Henriksen C, Slettahjell HB. Anthropometrics, diet, and resting energy expenditure in Norwegian adults with achondroplasia. Am J Med Genet A. 2019;179:1745–55.
4. Kanthimathinathan VS, Dockins J, Richardson N, et al. Morbidly obese achondroplasic and bariatric surgery [Abstract P450]. Surg Endosc. 2012;26(suppl 1):224–42.
